# Defect closure with endoscopic suturing improves endoscopic full-thickness resection of duodenal gastrointestinal stromal tumors

**DOI:** 10.1055/a-2072-3546

**Published:** 2023-05-04

**Authors:** Chu-Kuang Chou, Chien-Chuan Chen, Chi-Ming Tai, Kun-Feng Tsai, Chung-Ying Lee, Ding-Ek Toh, Sheng-Shih Chen

**Affiliations:** 1 Division of Gastroenterology and Hepatology, Department of Internal Medicine, Ditmanson Medical Foundation Chia-Yi Christian Hospital, Chiayi, Taiwan; 2 Obesity Center, Ditmanson Medical Foundation Chia-Yi Christian Hospital, Chiayi, Taiwan; 3 Internal Medicine, National Taiwan University Hospital, Taipei, Taiwan; 4 Division of Gastroenterology and Hepatology, Department of Internal Medicine, E-Da Hospital, I-Shou University, Kaohsiung, Taiwan; 5 School of Medicine for International Students, College of Medicine, I-Shou University, Kaohsiung, Taiwan; 6 Gastroenterology and Hepatology Section, Department of Internal Medicine, An Nan Hospital, China Medical University, Tainan, Taiwan; 7 Department of Medical Sciences Industry, Chang Jung Christian University, Tainan, Taiwan; 8 Division of Gastroenterology and Hepatology, Department of Internal Medicine, Shuang Ho Hospital, Taipei Medical University, New Taipei, Taiwan; 9 Division of Gastroenterology and Hepatology, Department of Internal Medicine, School of Medicine, College of Medicine, Taipei Medical University, Taipei, Taiwan; 10 TMU Research Center for Digestive Medicine, Taipei Medical University, Taipei, Taiwan; 11 Division of Gastroenterology and Hepatology, Department of Internal Medicine, Taipei Medical University Hospital, Taipei, Taiwan; 12 Trauma and Metabolic and Bariatric Center, Kaohsiung Veterans General Hospital, Kaohsiung, Taiwan

Endoscopic resection of duodenal gastrointestinal stromal tumors (GISTs) is challenging with non-negligible complications 1 . Endoscopic full-thickness resection (EFTR) is usually required 2 but remains uncommon for unsatisfactory defect closure. Clip-based methods are challenging for closing large defects and can be improved with mucosal flap preservation, which is time-consuming 3 . The strong and reliable whole-layer approximation with endoscopic suturing can change the decision-making of EFTR 2 4 5 .

A 59-year-old man was referred for a 2-cm muscle-origin tumor in the duodenal bulb ( Fig. 1 , Video 1 ); the digging biopsy failed to confirm its nature. Initially, EFTR with flap preservation for clip defect closure was planned. The resection was partially facilitated with adjustable snare-based traction ( Fig. 2 ) via a snare from an additional gastroscope 3 5 . The traction gastroscope was retracted, and the traction force was adjusted via the snare shaft during EFTR. However, the flap preservation failed, and defect closure with clip-based methods would be problematic 3 . We faced the decision on whether or not to abort this EFTR before perforation. With the backup of endoscopic suturing, we abandoned the flap and carefully dissected a 2-cm tumor from the muscle layer into the retroperitoneum ( Fig. 3 ). The 3-cm defect was closed completely with OverStitch Sx (Apollo Endosurgery, Austin, Texas, USA) ( Fig. 4 , Fig. 5 ). The patient resumed his diet 2 days later and was discharged uneventfully 4 days after EFTR. Pathology revealed a GIST with R0 resection. It took 45 minutes to preserve the flap, 50 minutes to do EFTR without flap preservation, and 25 minutes to close the defect with Overstitch SX. We could reduce the procedure time by abandoning the flap preservation with endoscopic suturing. EFTR with defect closure by endoscopic suturing system for duodenal GISTs will be more efficient and reliable.

**Fig. 1:**
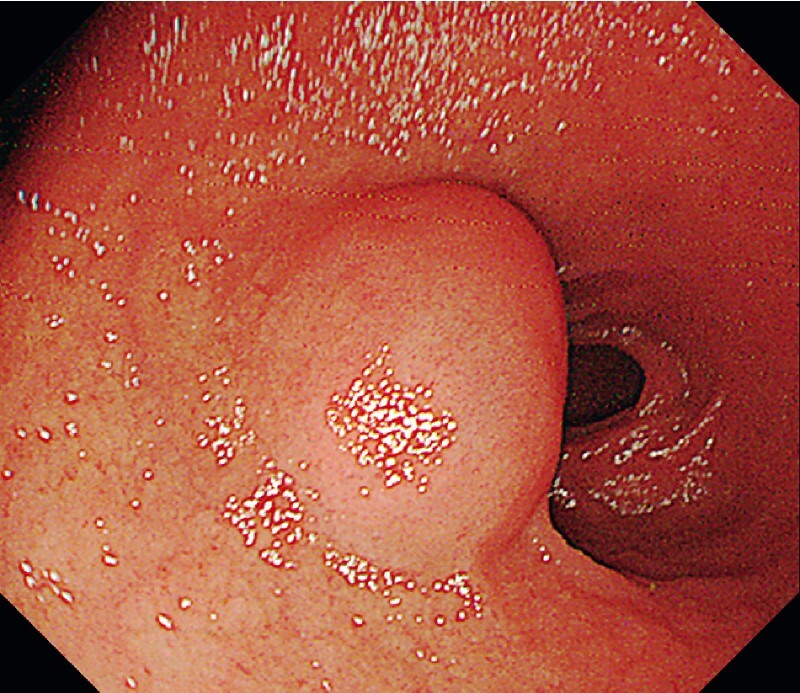
The tumor was located in the duodenal bulb.

**Video 1** Endoscopic suturing can rescue the defect of endoscopic full-thickness resection for a duodenal gastrointestinal stromal tumor.


**Fig. 2:**
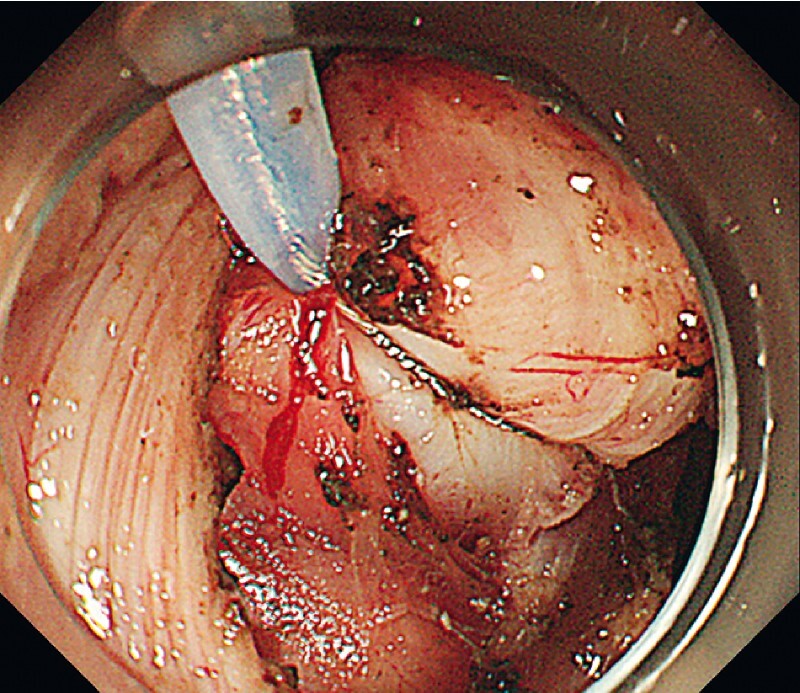
Endoscopic full-thickness resection (EFTR) was facilitated with snare-based traction.

**Fig. 3:**
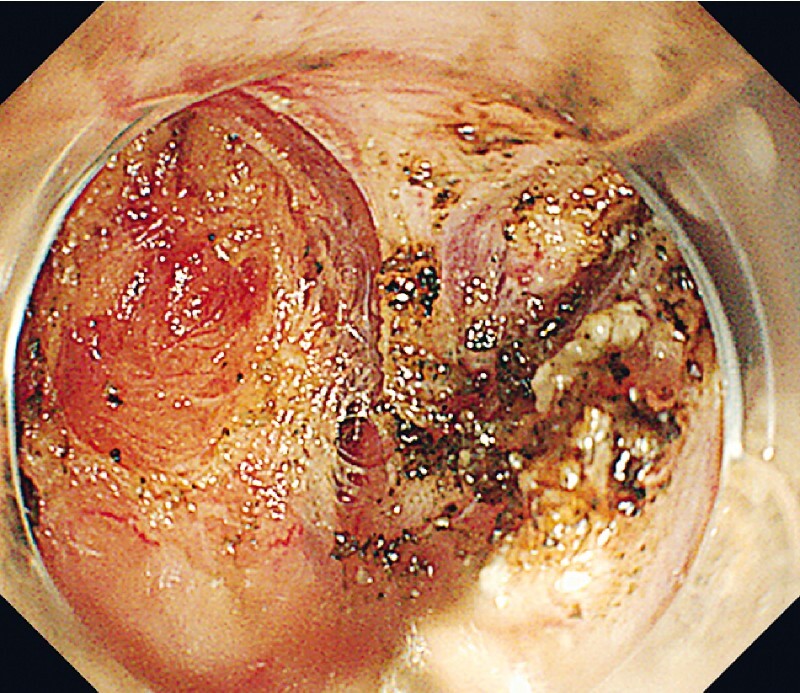
We dissected the tumor and defect into the retroperitoneum.

**Fig. 4:**
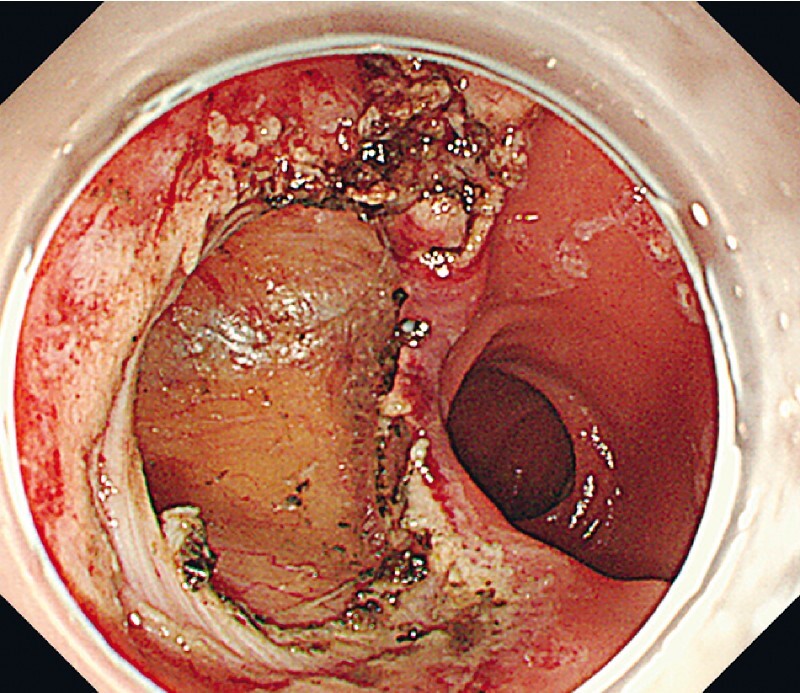
The perforation after EFTR was approximately 3 cm in size.

**Fig. 5:**
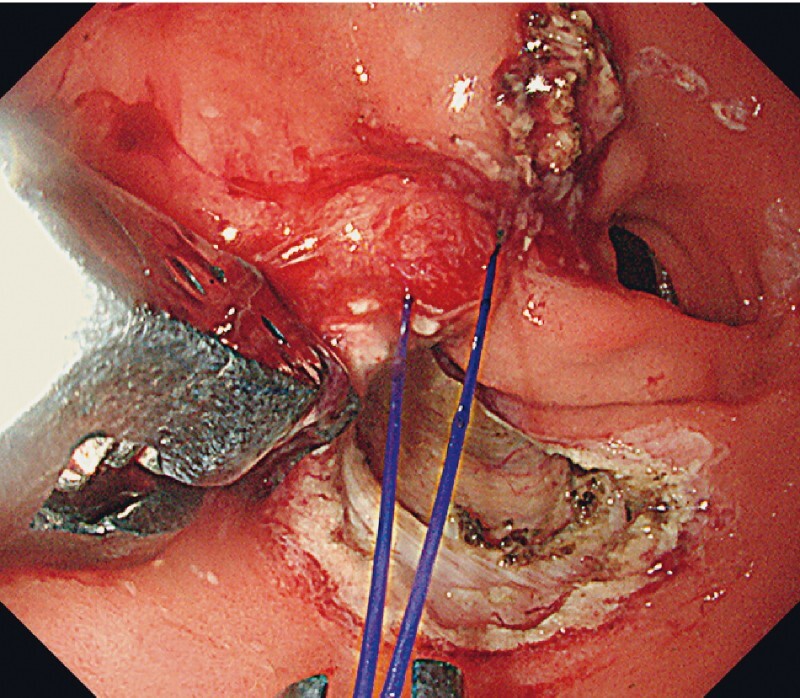
The endoscopic suturing system closed the defect completely.

Endoscopy_UCTN_Code_TTT_1AO_2AC

## Notes

Endoscopy E-Videos

https://eref.thieme.de/e-videos
*E-Videos* is an open access online section of the journal *Endoscopy* , reporting on interesting cases and new techniques in gastroenterological endoscopy. All papers include a high-quality video and are published with a Creative Commons CC-BY license. Endoscopy E-Videos qualify for HINARI discounts and waivers and eligibility is automatically checked during the submission process. We grant 100% waivers to articles whose corresponding authors are based in Group A countries and 50% waivers to those who are based in Group B countries as classified by Research4Life (see: https://www.research4life.org/access/eligibility/ ). This section has its own submission website at https://mc.manuscriptcentral.com/e-videos
